# Flexible MoS_2_ Anchored on Ge-Containing Carbon Nanofibers

**DOI:** 10.3390/nano13010075

**Published:** 2022-12-23

**Authors:** Meltem Yanilmaz, Jung Joong Kim

**Affiliations:** 1 Department of Textile Engineering, Istanbul Technical University, Istanbul 34469, Turkey; 2 Department of Civil Engineering, Kyungnam University, Changwon 51767, Republic of Korea

**Keywords:** germanium, carbon, nanofiber, batteries

## Abstract

Germanium is a promising anode material for sodium-ion batteries (SIBs) because of its high theoretical specific capacity, high ion diffusivity, and rate capability. However, large volume changes and pulverization deteriorate the cycling performance. In this study, flexible electrospun germanium/carbon nanofibers (Ge/CNFs) were prepared via electrospinning followed by heat treatment. MoS_2_ nanoparticles were subsequently anchored on the flexible Ge/CNFs via hydrothermal synthesis. Flexible MoS_2_ anchored on Ge/CNFs (MoS_2_@Ge/CNFs) was used as a self-standing binder-free anode in an SIB. Because of the high electronic conductivity of CNFs and the many active sites of MoS_2_ nanoparticles, a high initial capacity of over 880 mAh/g was achieved at a current density of 0.1 A/g. Moreover, the flexible binder-free MoS_2_@Ge/CNFs exhibited an excellent C-rate performance with a reversible capacity of over 300 mAh/g at a current density of 2 A/g. Therefore, we demonstrated that flexible binder-free MoS_2_@Ge/CNFs are a promising electrode candidate for a high-performance rechargeable battery.

## 1. Introduction

Renewable energy sources and energy storage systems have continued to attract research interests owing to growing concerns about climate change and other environmental problems. Lithium-ion batteries (LIBs) with high energy density, excellent rate capability, and a long cycle life are promising energy storage devices that can be used as a high-energy electrochemical power source; however, several drawbacks such as a lack of raw materials and safety risks limit their use [1,2]. Sodium-ion batteries (SIBs) offer an alternative to LIBs owing to the high availability and low cost of sodium [3,4]. However, the larger radius of Na^+^ (1.02 Å) compared with Li^+^ (0.76 Å) leads to slower diffusion kinetics and large volume expansion, and hence, poor electrochemical performance [5].

Germanium (Ge) is a promising anode material for SIBs owing to its high theoretical specific capacity (369 mAh/g), high ion diffusivity, and rate capability. However, the large volume expansion and pulverization of Ge during alloying and dealloying processes can result in a poor cycling performance [6,7]. Carbon materials are a promising matrix for loading active materials with high capacities in rechargeable batteries because of their low cost, good stability, excellent electronic conductivity, and tailorable structures. Nanostructured carbon can compensate for the volume change, prevent the aggregation of active materials, enhance the number of active sites, shorten ionic transport pathways, increase the electronic/ionic conductivity, and hence, improve the cycling and rate performance by facilitating fast electron/ion transmission [8]. Some Ge-based composites have been studied, including nanostructured carbon/Ge composites for LIBs [9,10,11,12,13]. However, there is a need to design and prepare Ge-containing composite anodes to improve their electrochemical properties. A promising fabrication method is to design hierarchical nanostructures with two or more active components. For instance, Mao et al. [14] prepared carbon@MoS_2_ with Sn nanoparticles and reported improved electrochemical properties with a high reversible capacity of 350 mAh/g.

MoS_2_ is a promising anode for SIBs because of its considerable theoretical capacity (673 mAh g^−1^) and large lattice spacing (0.62 nm). Moreover, the MoS_2_–carbon heterointerface offers fast diffusion of ions and electrons [14,15]. In this study, Ge was dispersed in flexible carbon nanofibers to improve the electronic conductivity and prevent agglomeration. Moreover, efficient ion and electron transport was maintained in the entire electrode without the use of binders or collectors owing to the excellent mechanical properties and flexibility of the germanium/carbon nanofibers (Ge/CNFs). Subsequently, the MoS_2_ nanoparticles were anchored on flexible Ge@CNFs. In this novel structure, Ge nanoparticles and MoS_2_ nanoparticles with high theoretical capacity improved the reversible capacity, whereas the flexible CNFs prevented the agglomeration of Ge and MoS_2_ and improved the conductivity. The MoS_2_ nanoparticles provided numerous active sites and improved the diffusion of ions and electrons. Moreover, the flexible nanofibrous carbon structure with a fine fiber diameter of approximately 200 nm improved the impregnation of electrolyte into the electrode structure, thereby improving the rate capability. Therefore, the novel electrode structure resulted in an enhanced reversible capacity and cyclic stability owing to the excellent electronic conductivity of the flexible CNFs and high number of active sites of the MoS_2_ nanoparticles. When the flexible MoS_2_ anchored on Ge/CNFs was used as the anode in SIBs, a high initial capacity of over 880 mAh/g was achieved at a current density of 0.1 A/g. Moreover, the flexible, binder-free MoS_2_@Ge/CNFs exhibited an excellent C-rate performance with a reversible capacity of over 300 mAh/g at a current density of 2 A/g. This approach provides a facile method to design high-performance electrodes for rechargeable batteries and supercapacitors.

## 2. Materials and Methods

Polyacrylonitrile (PAN), N,N-dimethylformamide, thiourea, and ammonium molybdate tetrahydrate were purchased from Sigma-Aldrich. PAN nanofibers were prepared by electrospinning; an 8 wt.% PAN solution was fed into the syringe, and a high voltage of 15 kV was applied to the solution. The spinneret-to-collector distance was 15 cm, and the feeding rate was 0.8 mL/h. To obtain flexible Ge/CNFs, a certain amount of Ge was added to the PAN solution before electrospinning, and the Ge-containing PAN nanofibers were stabilized at 280 °C for 3 h and carbonized at 800 °C in a nitrogen atmosphere. In addition, MoS_2_ was anchored to flexible Ge@CNFs via hydrothermal synthesis. A certain amount of thiourea and ammonium molybdate tetrahydrate was dissolved in distilled water, and the flexible Ge/CNFs were immersed in this solution. The solution was then transferred to a hydrothermal reactor and heated to 180 °C for 24 h.

Scanning electron microscopy (SEM; Zeiss Sigma 300, Oberkochen, Germany) was performed to examine the morphology of the flexible MoS_2_@Ge/CNFs. Energy dispersive X-ray (EDX; Thermo Scientifıc Apreo 2 S LoVac) mapping was performed to examine the distribution of the elements on the CNFs. X-ray diffraction (XRD; PANalytical Empyrean, Malvern, UK) pattern with a step of 0.01 and speed of 4º/min and Raman spectroscopy (WITech alpha 300R, Ulm, Germany) were used to characterize the structure of the flexible MoS_2_@Ge/CNFs. 

The as-prepared flexible MoS_2_@Ge/CNFs were used as anodes in sodium ion cells. The electrochemical performance was measured using CR2032 type coin cells. The flexible MoS_2_@Ge/CNF electrodes were cut into a 12-mm diameter disk and then used as the working electrode without additional metal current collectors, conductive additives, or polymer binders. The counter/reference electrode was sodium metal (Na, Sigma); a glass fiber film (Whatman GF/D) was used as the separator and the electrolyte was a solution of 1 M NaClO_4_ in ethylene carbonate (EC)/propylene carbonate (PC) with a volume ratio of 1:1, with 5% fluorinated ethylene carbonate (FEC). Galvanostatic measurements were conducted on battery testing systems (Hefa battery analyzer) with a potential range of 0.0−2.5 V (vs. Na/Na+).

## 3. Results

**Figure 1:**
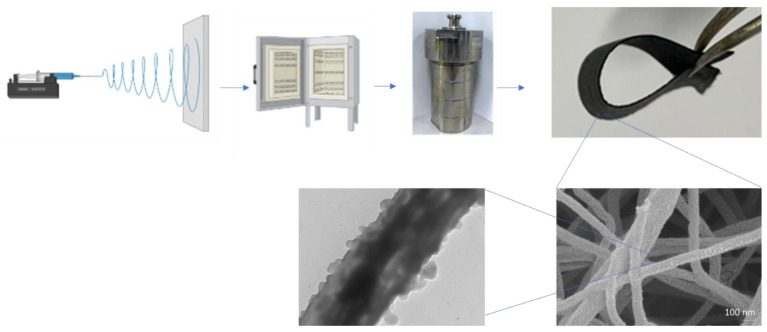
Schematic diagram of the preparation of the flexible MoS_2_@Ge/CNFs.

**Figure 2:**
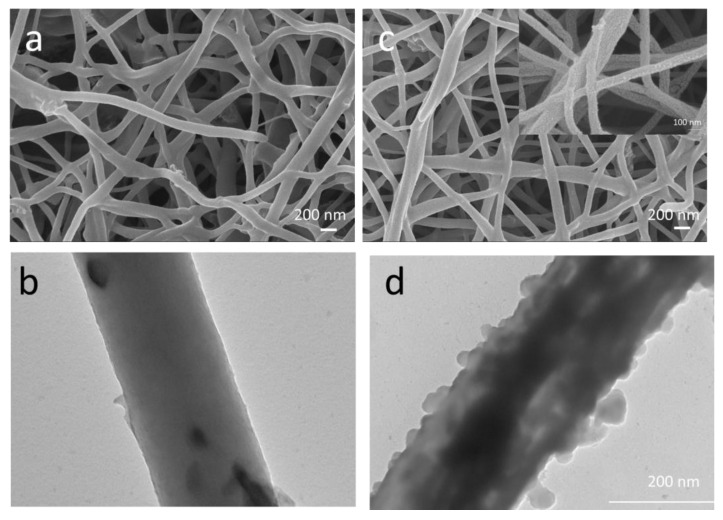
SEM and TEM images of the flexible Ge/CNFs (**a**,**b**) and MoS_2_@Ge/CNFs (**c**,**d**).

A schematic diagram of the preparation of the flexible MoS_2_@Ge/CNFs is shown in Figure 1. Flexible MoS_2_@Ge/CNFs were prepared using a facile approach that combines electrospinning, heat treatment, and hydrothermal synthesis. The photograph of the MoS_2_@Ge/CNFs is shown in Figure 1 to depict the flexibility. The SEM images of the flexible Ge/CNFs and MoS_2_@Ge/CNFs are shown in Figure 2. The average fiber diameter is approximately 200 nm for the Ge/CNFs, and nano-sized MoS_2_ anchoring does not significantly affect the average fiber diameter. 

The TEM images shown in Figure 2 depict the MoS_2_ nanoparticle coating on the flexible Ge/CNFs. Carbon nanofibers with thin fiber diameters and rough surfaces are reported to be effective for improving electronic and ionic transport. Moreover, carbon nanofibers with fine fiber diameters additionally improve the active sites in the electrode structure. The nano-sized active materials on carbon nanofibers increase the ion transport rates in rechargeable batteries [16,17].

**Figure 3:**
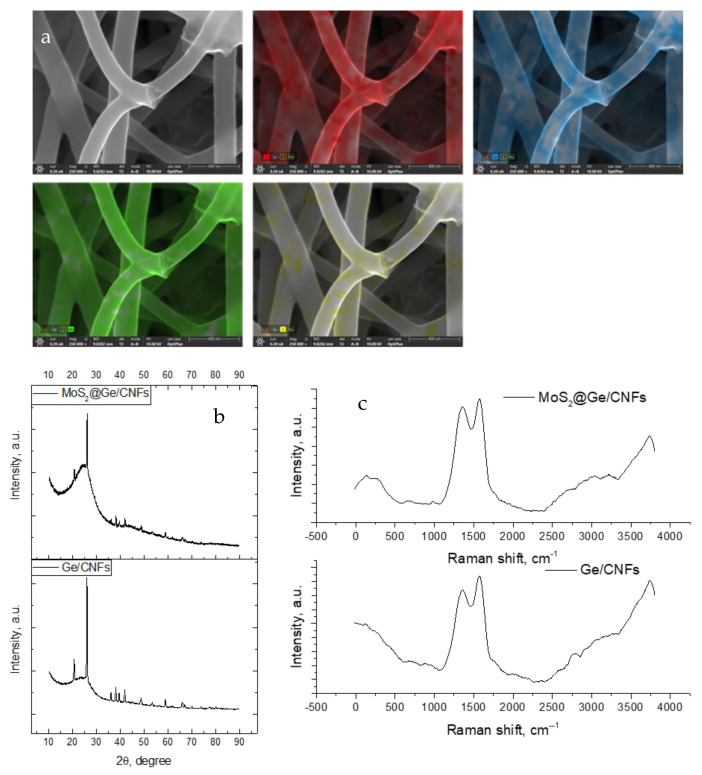
(**a**) EDX mapping and spectrum of the flexible MoS_2_@Ge/CNFs; (**b**) XRD pattern and (**c**) Raman spectra of the flexible Ge/CNFs and MoS_2_@Ge/CNFs.

The EDX mapping of the flexible MoS_2_@Ge/CNFs as well as the corresponding SEM image are shown in Figure 3a. The figure additionally shows uniform distributions of the MoS_2_ coating and Ge nanoparticles. According to EDX data, carbon, MoS_2_ and Ge contents are 65%, 25% and 10%, respectively. Figure 3b shows the XRD patterns of the Ge/CNFs. The 2θ peaks at approximately 27°, 45°, 53°, 66°, and 73° are ascribed to the (111), (220), (311), (400), and (331) lattice planes of germanium, respectively. A peak at around 25° is linked to the (002) layers of graphite structure [18,19]. In the XRD pattern of the flexible MoS_2_@Ge/CNFs, the peaks at 39.5° and 58° are respectively linked to the (103) and (110) planes of MoS_2_ [3]. In addition, the peaks of MoS_2_ are weak and broad because of their nanosized or amorphous structure [20,21].

Figure 3c shows the Raman spectra of the Ge/CNFs and MoS_2_@Ge/CNFs. The two peaks at approximately 1355 cm^−1^ and 1585 cm^−1^ correspond to the D and G bands of carbon, respectively. The D band is related to the defect-induced breathing mode of sp2 rings, whereas the G band corresponds to the first-order scattering of the E2g mode of the sp^2^ domains. The ID/IG ratio was used to estimate the degree of disorder of the graphitized structure. The ID/IG ratios for the Ge/CNFs and flexible MoS_2_@Ge/CNFs are approximately 1.0, thereby confirming that the structure is highly disordered with many defects [9]. 

**Figure 4:**
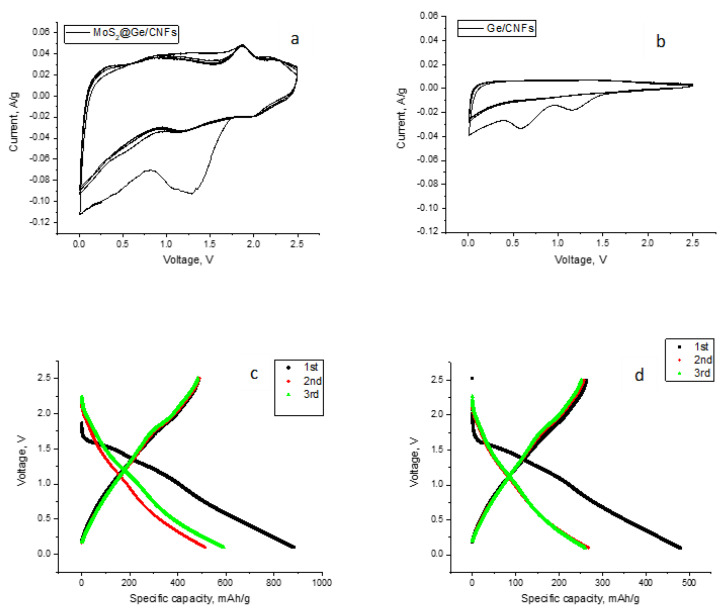
First cycle CV curves and charge–discharge curves of: (**a**,**c**) MoS_2_@Ge/CNFs and (**b**,**d**) Ge/CNFs.

Figure 4a shows the initial cyclic voltammetry curves of the flexible MoS_2_@Ge/CNFs at 0.01–2.5 V with a scan rate of 0.2 mV s^−1^. In the first cathodic scan, the broad reduction peak at approximately 1.1 V corresponds to the insertion of Na^+^ into the interlayers of MoS_2_ (MoS_2_ + xNa + + xe− → NaxMoS_2_) and the irreversible generation of the solid–electrolyte interphase (SEI) film. In the first oxidation scan, an outstanding anodic peak was observed at approximately 1.75 V as a result of the oxidation of Mo to MoS_2_ [14,22]. The large irreversibility in the first cycle can be ascribed to the formation of an SEI layer. The overlapping of the following cycles demonstrates the good reversibility of the MoS_2_@Ge/CNF electrodes. The CV curves of the Ge/CNFs are also shown in Figure 4b for comparison. In the first cycle, the reduction peaks in the cathodic scan at approximately 0.65 V and 1.2 V can be attributed to the reaction between Na^+^ and Ge (Na_x_Ge <=>Ge + x Na+ + x e^-^) and the SEI formation, respectively. [6,23]. Two peaks corresponding to the alloying reaction and SEI formation were also seen in CV curves of Ge electrodes [24,25]. There are no apparent redox peaks in the anodic and cathodic scans, and the current is nearly on the reduction branch, which indicates that the SEI is being built up on the surface of the electrode. A similar CV curve was also reported for the Ge/graphene electrode [17].

Figure 4c shows the charge–discharge curves of the first three cycles of the flexible MoS_2_@Ge/CNF electrode at 0.1 A g^−1^. A large irreversible capacity was observed in the first cycle because of SEI formation, which is consistent with the CV curve. The charge–discharge curves of the second and third cycles almost overlap, indicating the good reversibility of the flexible MoS_2_@Ge/CNF electrode. The initial specific discharge and charge capacities of the flexible MoS_2_@Ge/CNFs are 883 mAh/g and 487 mAh/g at 0.1 A/g, respectively, corresponding to an initial Coulombic efficiency of approximately 55%. In the second and third cycles, the Coulombic efficiencies are 95% and 99%, respectively.

The first charge–discharge curves of the flexible Ge/CNF electrodes are also shown in Figure 4d for comparison. The initial discharge and charge capacities are 478 mAh/g and 263 mAh/g, respectively. The nearly overlapping charge and discharge curves observed in subsequent cycles indicate the excellent reversibility of the flexible Ge/CNFs.

**Figure 5:**
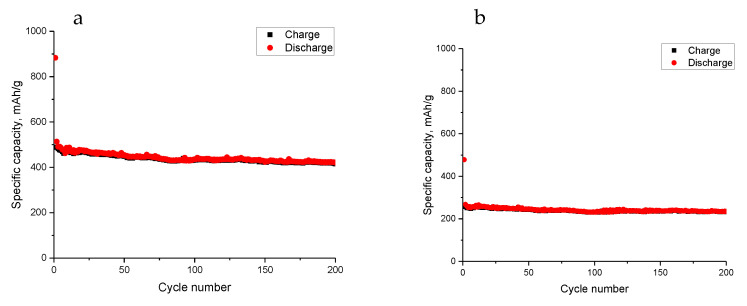
Cycling performance of: (**a**) flexible MoS_2_@Ge/CNFs and (**b**) Ge/CNFs.

Figure 5 shows the cycling performance of the flexible MoS_2_@Ge/CNF electrode. The cycling performance of the flexible Ge/CNF electrodes is also shown. A high reversible capacity of more than 400 mAh/g was achieved over 200 cycles when the flexible MoS_2_@Ge/CNF electrode was used. The nano-sized MoS_2_ layer improved the reversible capacity, and the flexible fibrous carbon structure improved the electronic conductivity of the binder-free electrodes, compensated for the volume change, and increased the number of active sites. 

Chen et al. [22] fabricated MoS_2_@SnO_2_@C via hydrothermal synthesis and the reversible capacity of approximately 400 mAh/g was reached in 150 cycles and the high electrochemical results were ascribed to the sandwich-like structure. However, the reversible capacity of the flexible Ge/CNFs was only approximately 200 mAh/g. A similar reversible capacity of approximately 200 mAh/g was also reported by Liu et al. [26] for Ge-containing carbon, and the stable cycling performance was attributed to the amorphous dual carbon composite, which compensated for the volume expansion and provided an additional capacitance contribution, resulting in a stable reversible capacity. In another study, Liu et al. [6] prepared Ge-containing CNF electrodes and reported a reversible capacity of 160 mAh/g. 

**Figure 6:**
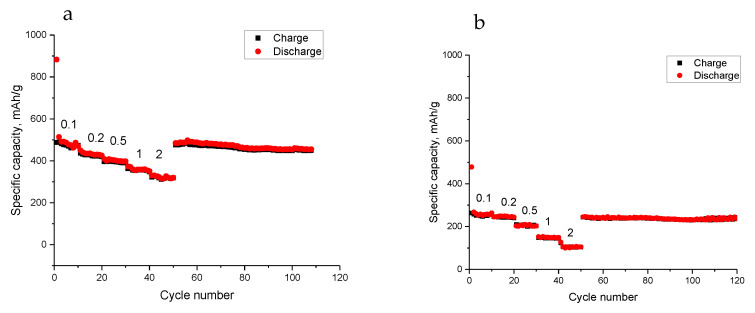
C-rate performance of the flexible (a) MoS_2_@Ge/CNFs and (**b**) Ge/CNFs.

Figure 6 shows the rate performance of the flexible MoS_2_@Ge/CNF electrode. A discharge specific capacity of 480 mAh/g was maintained at 0.1 A/g, and the discharge capacities are approximately 430 mAh/g, 400 mAh/g, 360 mAh/g, and 320 mAh/g at current densities of 0.2 A/g, 0.5 A/g, 1 A/g, and 2 A/g, respectively. Chen et al. [27] prepared MoS_2_/CNF via electrospinning and the composite electrode only delivered a capacity of around 240 mAh/g at 1 C. Moreover, the reversible capacity was approximately 480 mAh/g when the current density was reversed back to 0.1 A/g. These well-maintained capacities at various current densities demonstrate the excellent conductivity of the flexible MoS_2_@Ge/CNF electrode, resulting in high utilization and excellent rate capability of the material. In Figure 6b, the C rate performances of Ge/CNFs electrodes are presented. The reversible capacities are around 260, 246, 210, 149 and 105 mAh/g, respectively, at the current densities of 0.1, 0.2, 0.5, 1 and 2 mA/g. Liu et al. [6] prepared Ge containing CNFs via electrospinning and the reversible capacities of around 210 and 160 mAh/g were reported at the current densities of 0.1 and 0.5 mA/g whereas flexible Ge/CNFs delivered capacities of around 260 and 210 mAh/g at the same current densities. Wang et al. [17] fabricated Ge@graphene electrodes and reported reversible capacities of around 200, 150, and 50 mAh/g at the current densities of 0.1, 0.2 and 1 A/g, respectively. As can be seen, the flexible Ge/CNFs electrode has better capacities owing to the superior mechanical stability of CNFs. In another study, a SnS_2_@MoS_2_@rGO electrode delivered a reversible capacity of 400 mAh/g at 0.2 A/g [15]. Sn doped MoS_2_/carbon electrodes delivered a discharge capacity of around 400 mAh/g at 1 A/g [28]. Zheng at al [29] prepared Sn-MoS_2_-C@C Microspheres with a reversible capacity of 360 at 1A/g. Mao et al. [14] prepared MoS_2_/Sn/carbon electrodes and the reversible capacity was found as 300 mAh/g at 2 A/g whereas flexible MoS_2_@Ge/CNFs delivered a reversible capacity of 320 mAh/g at the high current rate of 2 A/g. Mao et al. [14] prepared MoS_2_/Sn/carbon electrodes and the reversible capacity was found as 300 mAh/g at 2 A/g whereas flexible MoS_2_@Ge/CNFs delivered a reversible capacity of 320 mAh/g at the high current rate of 2 A/g. This high performance of flexible MoS_2_@Ge/CNFs at high current densities could be ascribed to a high number of active sites created by MoS_2_ anchoring on flexible CNFs with thin fiber diameters. The outstanding electrochemical properties of the flexible MoS_2_@Ge/CNF electrode are ascribed to its unique nanostructure. Nano-sized MoS_2_ anchoring on flexible Ge/CNF electrodes resulted in an enhanced reversible capacity with a stable cycling performance. The results can be attributed to the evenly distributed MoS_2_ nanoparticles on the flexible Ge@CNFs, which provide many active sites for sodium ion intercalation and a nanofibrous flexible carbon structure with fine fiber diameters of approximately 200 nm, which led to improved ion and electron diffusion, and hence, improved rate performance.

## 4. Conclusions

Flexible MoS_2_ anchored on Ge/CNFs was successfully prepared by electrospinning, heat treatment, and hydrothermal synthesis. Flexible CNFs with fine fiber diameters of approximately 200 nm accommodate the volume expansion of Ge nanoparticles and provide more active sites, thereby improving the specific capacity and cyclic stability of binder-free electrode materials. MoS_2_ further improved the reversible capacity and C-rate performance owing to its high theoretical capacity and nano-sized anchoring on the flexible CNFs. In addition, the fibrous structure facilitates the fast transfer of ions and electrons and ensures that the material exhibits excellent cyclic stability and rate performance. This facile approach can provide a platform for designing nanostructured electrode materials for high-performance rechargeable batteries and supercapacitors.

## Data Availability

Not applicable.
